# Whole-Genome Sequence of a Brucella pinnipedialis Sequence Type 54 Strain Isolated from a Hooded Seal (*Cystophora cristata*) from the North Atlantic Ocean, Norway

**DOI:** 10.1128/MRA.00271-21

**Published:** 2021-05-06

**Authors:** Michel S. Zygmunt, Gilles Vergnaud, Axel Cloeckaert

**Affiliations:** a INRAE, UMR ISP, Université de Tours, Nouzilly, France; b Université Paris-Saclay, CEA, CNRS, Institute for Integrative Biology of the Cell, Gif-sur-Yvette, France; Georgia Institute of Technology

## Abstract

Since the 1990s, Brucella strains have been isolated from a wide variety of marine mammal species. We report the first complete genome sequence of a Brucella strain isolated from a hooded seal (*Cystophora cristata*), Brucella pinnipedialis strain 23a-1 of sequence type 54, found in the North Atlantic Ocean surrounding Norway.

## ANNOUNCEMENT

Brucella constitutes a genus comprising pathogenic species affecting livestock and causing disease in humans, of which the main representative, in terms of global health impact, is Brucella melitensis (1). Since the 1990s, a number of new species, mostly from wildlife, have been identified. Among them are the species Brucella ceti and Brucella pinnipedialis isolated from marine mammal species, with cetaceans and pinnipeds, respectively, as preferred hosts (2). These species could be further subdivided into a large number of subtypes by genotyping. In particular, multilocus sequence typing (MLST) and multiple-locus variable-number tandem repeats (VNTR) analysis (MLVA) proved to be highly discriminatory (3–5). The latest MLST21 scheme (5) identified over 100 Brucella sequence types (STs), including 16 STs for marine mammal brucellae. Similarly, MLVA16 identified seven major clusters of strains within B. ceti and B. pinnipedialis, with distinct MLVA16 genotypes in each cluster (3). Some clusters appeared to be restricted to specific marine mammal hosts. For example, MLVA16 cluster C3 genotypes are restricted to a single seal species host, i.e., hooded seal (Cystophora cristata). In MLST21, hooded seal isolates currently constitute two STs, ST53 and ST54 (5). The hooded seals form two populations, the Northeast and Northwest Atlantic populations, and their specific biology, physiology, habitat, and behavior, relative to those of other seal species, have been reviewed by Nymo et al. (6).

Among hooded seal strains previously typed by MLVA16 (3), we selected for whole-genome sequencing (WGS) B. pinnipedialis strain 23a-1, constituting a single specific genotype in the MLVA16 C3 cluster (https://microbesgenotyping.i2bc.paris-saclay.fr/databases/public) (3). This strain was isolated from a hooded seal found in the Northeast Atlantic Ocean in Norway (3). The genomic DNA was extracted from the trypticase soy agar with 0.6% yeast extract (TSAYE)-cultured strain with the DNeasy blood and tissue kit (Qiagen, Les Ulis, France). WGS and library construction were performed by GenoScreen (Lille, France), using the PacBio Sequel platform. The sequencing libraries were generated with a SMRTbell template preparation kit v3.0. SMRT Link (v5.1.0.26412) was used for quality control and filtering, which resulted in 127,654 subreads with an average length of 6,868 bp and an *N*_50_ value of 8,281 bp. The assembly was circularized by Circlator v1.5.5 using the raw reads corrected by Canu v1.6. Default parameters were applied for all software packages. The genome sequence was annotated by the NCBI Prokaryotic Genome Annotation Pipeline v4.13 (7).

The whole genome of strain 23a-1 consisted of two circular chromosomes, with sizes of 2,120,314 bp and 1,192,109 bp and with a deduced G+C content of 57.3%, as seen for other Brucella species. WGS confirmed the specific MLVA16 genotype of strain 23a-1, and the deduced ST in MLST21 was ST54. The PacBio technology also allowed identification of the distribution of the genus-specific insertion sequence IS*711* (8). A total number of 32 IS*711* elements were identified, dispersed on both chromosomes. In addition, the lack of a 67-kb DNA fragment flanked by IS*711* elements located on chromosome 2, relative to B. pinnipedialis strains from other seal species (9), was confirmed.

### Data availability.

The complete genome sequence of B. pinnipedialis strain 23a-1 can be retrieved from the European Nucleotide Archive within BioProject PRJNA664313, with assembly accession number GCA_015624465 (accession number CP061816.1 for chromosome 1 and accession number CP061815.1 for chromosome 2). The raw sequencing results were deposited in the NCBI SRA database under accession number SRR14150359.
